# Supplementary material for: Dynamic transcriptomic profiles of zebrafish gills in response to zinc supplementation
Source: BMC Genomics. 2010 Oct 11;11:553. doi: 10.1186/1471-2164-11-553 (PMC3091702; doi:10.1186/1471-2164-11-553)
Supplement: Additional file 2 — Interactive Direct Interaction Network representing the molecular interactions between zinc, copper, iron, calcium and proteins encoded by transcripts changed by zinc supplementation. Mini web-site containing index.html and hyperlinked pages in subdirectory describing a Direct Interaction Network automatically generated based on curated interactions contained within the proprietary PathwayArchitect database. Ovals represent proteins and the circles symbolize metal ions. Objects are coloured by their abundance in zebrafish at the time-point they were significantly different from the control is a scale from -4 fold (dark green) to +4 fold (dark red). Where significant differences were found at more than one time-point, the colour overlay shows expression at the first instance. Dark blue squares denote 'binding', and light blue squares 'expression'; green squares stand for 'regulation', green diamonds for 'metabolism', and green circles for 'promoter binding'. Arrow heads indicate directionality of the interaction where annotated. All nodes and edges can be further interrogated by selecting the relative area of the image. [file 1471-2164-11-553-S2.zip › PathwayArchitect Zn xs DIN/784548.html]

# REGULATION:

|  |  |
| --- | --- |
| Type | REGULATION |
| Effect | Negative |


---

|  |  |
| --- | --- |
| Score | 0 |


---

|  |  |
| --- | --- |
| Reference Count | 7 |


---

|  |  |
| --- | --- |
| Mechanism | Unknown |


---

|  |  |
| --- | --- |
| Reference:0 || Sentence | "Dietary iron deficiency in +/+ and +/hpx mice decreased tibia iron concentrations but had no effect on tibia calcium and phosphorus concentrations or femur structural or mechanical properties." |
| PMID | 10703928 |
| Year | 2000 |
| Species | Mouse |
| Journal | J Bone Miner Res |
| RefScore | 0 |
| Source | PArchNLP |
  |
|  |  |


---

|  |  |
| --- | --- |
 Reference:1 || Sentence | "This Hp mouse line provides an opportunity to study the effects of transferrin deficiency on development and iron homeostasis." |
| PMID | 2752125 |
| Year | 1989 |
| Species | Mouse |
| Journal | Blood |
| RefScore | 1 |
| Source | PArchNLP |
  ||  |  |


---

|  |  |
| --- | --- |
 Reference:2 || Sentence | "The hypoferremic response in C57 black mice during EL4 lymphoma cell growth was followed to determine if a lowered transferrin iron saturation inhibited iron acquisition and growth by these tumor cells." |
| PMID | 3435663 |
| Year | 1987 |
| Species | Mouse |
| Journal | Biochem Cell Biol |
| RefScore | 1 |
| Source | PArchNLP |
  ||  |  |


---

|  |  |
| --- | --- |
 Reference:3 || Sentence | "Transferrin levels and serum iron levels in aging humans also diminish, as observed from measurements of total iron binding capacity and percent iron saturation in sera from 701 individuals ranging from 0 to 99 years of age." |
| PMID | 1382606 |
| Year | 1992 |
| Species | Human |
|  | Mouse |
| Journal | Biochim Biophys Acta |
| RefScore | 1 |
| Source | PArchNLP |
  ||  |  |


---

|  |  |
| --- | --- |
 Reference:4 || Sentence | "The present findings demonstrate that iron distribution in the brain is changed by transferrin deficiency and that iron abnormally accumulates in the brain of HP mice." |
| PMID | 11532431 |
| Year | 2001 |
| Species | Mouse |
| Journal | Brain Res |
| RefScore | 2 |
| Source | PArchNLP |
  ||  |  |


---

|  |  |
| --- | --- |
 Reference:5 || Sentence | "However, in the absence of Tf in the media, significantly more iron was retained in the cells than if Tf were present in both normal and Hp glial cultures. 54Mn release was minimally affected by extracellular Tf." |
| PMID | 9514199 |
| Year | 1998 |
| Species | Mouse |
| Journal | J Neurosci Res |
| RefScore | 2 |
| Source | PArchNLP |
  ||  |  |


---

|  |  |
| --- | --- |
 Reference:6 || Sentence | "Finally, when a monoclonal antibody to the mouse transferrin receptor and iron-nitriotriacetate were substituted for iron-transferrin in serum-free medium, proliferation was reduced by greater than 95%." |
| PMID | 3002966 |
| Year | 1986 |
| Species | Mouse |
|  | Human |
| Journal | Immunology |
| RefScore | 1 |
| Source | PArchNLP |
  |


---

|  |  |
| --- | --- |
